# A predictive model for assessing prognostic risks in gastric cancer patients using gene expression and methylation data

**DOI:** 10.1186/s12920-020-00856-0

**Published:** 2021-01-06

**Authors:** Dan Luo, QingLing Yang, HaiBo Wang, Mao Tan, YanLei Zou, Jian Liu

**Affiliations:** 1 Department of General Surgery, Chengdu Fifth People’s Hospital, 33 Mashi St, Chengdu, 610000 Sichuan China; 2 Department of Pulmonary and Critical Care Medicine, Chengdu Fifth People’s Hospital, 33 Mashi St, Chengdu, 610000 Sichuan China

**Keywords:** Gastric cancer, Methylation, Prognosis, Differential expression, Biomarker

## Abstract

**Background:**

The role(s) of epigenetic reprogramming in gastric cancer (GC) remain obscure. This study was designed to identify methylated gene markers with prognostic potential for GC.

**Methods:**

Five datasets containing gene expression and methylation profiles from GC samples were collected from the GEO database, and subjected to meta-analysis. All five datasets were subjected to quality control and then differentially expressed genes (DEGs) and differentially expressed methylation genes (DEMGs) were selected using MetaDE. Correlations between gene expression and methylation status were analysed using Pearson coefficient correlation. Then, enrichment analyses were conducted to identify signature genes that were significantly different at both the gene expression and methylation levels. Cox regression analyses were performed to identify clinical factors and these were combined with the signature genes to create a prognosis-related predictive model. This model was then evaluated for predictive accuracy and then validated using a validation dataset.

**Results:**

This study identified 1565 DEGs and 3754 DEMGs in total. Of these, 369 were differentially expressed at both the gene and methylation levels. We identified 12 signature genes including *VEGFC*, *FBP1*, *NR3C1*, *NFE2L2*, and *DFNA5* which were combined with the clinical data to produce a novel prognostic model for GC. This model could effectively split GC patients into two groups, high- and low-risk with these observations being confirmed in the validation dataset.

**Conclusion:**

The differential methylation of the 12 signature genes, including *VEGFC*, *FBP1*, *NR3C1*, *NFE2L2*, and *DFNA5*, identified in this study may help to produce a functional predictive model for evaluating GC prognosis in clinical samples.

## Background

Gastric cancer (GC) is the fifth-most common cancer in the world and is associated with high mortality and dismal prognosis as a result of its delayed diagnosis [1, 2]. There are approximately 1 million new cases of GC diagnosed every year and the mortality is about 720,000 deaths per year worldwide [3]. In western countries, the mortality for GC is extremely high because diagnostic screening approaches are insufficient and most patients (≥ 50%) are only diagnosed at later stages [2]. Understanding the underlying pathogenesis of GC has facilitated the identification of novel molecular biomarkers, which researchers hope will help to advance the diagnosis of this disease at earlier stages.

Many genetic and epigenetic events have been linked to carcinogenesis. The major hallmarks of any epigenetic events include alterations at the promoter CpG sites within the gene or changes to the histone at the chromatin level, and the most widely studied and best characterised epigenetic events include differential methylation of tumour suppressors and oncogenes [4].

In GC, multiple epigenetic modifications have been linked to disease progression, and these alterations may contribute to the identification of biomarkers for early diagnosis [4]. Using epigenome wide and gene-specific DNA methylation analyses, a DNA methylation biomarker panel, which includes *IRF4*, *ELMO1*, *CLIP4*, and *MSC,* has been found to link GC and gastritis, and this panel has been shown to be useful in endoscopic biopsies allowing for the earlier detection of GC in these samples [5]. In a study of the Chinese population *COX-2* methylation levels were shown to be reduced in the anti-*Helicobacter pylori* intervention group, compared with the placebo group, indicating that this differential methylation might be a useful indicator of chemoprevention efficacy for GC [6]. The aberrant methylation of the tumour suppressor gene, *FAT4*, in peripheral blood leukocytes has been linked to increased GC risk [7]. While decreased expression of *HOXB13*, caused by methylation of its promoter, is a reliable marker for poor prognosis in GC [8]. Increased methylation of the *LINE1* and *IGF2* differentially methylated regions (DMRs) correlates with more aggressive GC phenotypes and thus are considered potential biomarkers for GC progression [9].

Despite these encouraging findings, the underlying gene methylation mechanisms used in GC remain obscure. In addition, evaluation of methylation in samples collected in previous studies remains relatively rare. Therefore, we searched GC-related gene and methylation expression profiles found in public databases, and combined these data using meta-analysis, to enlarge the sample size and enhance statistical power. Using a series of bioinformatics tools and survival analysis, we could reveal the association between gene methylation and GC prognosis identifying several novel prognostic biomarkers.

## Methods

### Data resource and sample classification

#### Dataset for meta-analysis

The gene expression and methylation profile datasets were selected from the GEO database (http://www.ncbi.nlm.nih.gov/geo/), using the keywords “gastric cancer” and “Homo sapiens”. The inclusion criteria for the datasets were as follows: (1) the dataset must include both gastric cancer tumour samples and normal tissue samples; and (2) have at least 50 samples in the dataset. Five eligible gene expression and two methylation profile datasets were identified and downloaded. GSE26942 was from the GPL6947 platform and consisted of 205 gastric tumour tissue samples (GC samples) and 12 gastric normal tissue samples (normal samples) (https://www.ncbi.nlm.nih.gov/geo/query/acc.cgi?acc=GSE26942). GSE29727, which included 134 GC samples and 134 normal samples, (https://www.ncbi.nlm.nih.gov/geo/query/acc.cgi?acc=GSE29727) was isolated from GPL96. GSE54129, which included 111 GC samples and 21 normal samples, (https://www.ncbi.nlm.nih.gov/geo/query/acc.cgi?acc=GSE54129) and GSE64951, which had 63 GC samples and 31 normal samples, (https://www.ncbi.nlm.nih.gov/geo/query/acc.cgi?acc=GSE64951) were both from GPL570. GSE65801 was from the GPL14550 and consisted of 64 samples made up of 32 GC and 32 normal tissues (https://www.ncbi.nlm.nih.gov/geo/query/acc.cgi?acc=GSE65801). Both of the methylation datasets were identified from the GPL8490 platform, GSE25869 comprised 74 samples (GC sample, n = 42; normal sample, n = 32, https://www.ncbi.nlm.nih.gov/geo/query/acc.cgi?acc=GSE25869), and GSE30601 had 297 samples (GC sample, n = 203; normal sample, n = 94, https://www.ncbi.nlm.nih.gov/geo/query/acc.cgi?acc=GSE30601). The attributes for each of these datasets are summarised in Table 1. Detailed clinical information for each of the samples in these datasets was collected from the data derived in the GEO database.

**Table 1:** Information of gene expression and methylation profiles in the datasets included in the meta-analysis

GEO accession		Platform	Total sample number	Normal	Cancer
Gene expression					
GSE26942		GPL6947	217	12	205
GSE29727		GPL96	268	134	134
GSE54129		GPL570	132	21	111
GSE64951		GPL570	94	31	63
GSE65801		GPL14550	64	32	32
Gene methylation					
GSE25869		GPL8490	74	32	42
GSE30601		GPL8490	297	94	203

#### Predictive modelling dataset

Relative gastric cancer gene expression and methylation profiles were downloaded from The Cancer Genome Atlas (TCGA, https://gdc-portal.nci.nih.gov/) database. Then, the gene expression and gene methylation profiles were matched. This created a single dataset containing a total of 398 matched tumour samples. Of these, 360 samples had complete prognostic information. These data were then used as the training dataset for the predictive model for prognosis developed in our study. Another set of gastric cancer-related gene expression profiles, GSE62254, was then downloaded from the GEO database (https://www.ncbi.nlm.nih.gov/geo/query/acc.cgi?acc=GSE62254). This dataset was from the GPL570 [HG-U133_Plus_2] Affymetrix Human Genome U133 Plus 2.0 Array platform and included the data from.300 gastric cancer tumour tissue samples, and was used as an independent validation dataset. Patients’ clinical characteristics are listed in Table 2. The mean age of the patients was 64.9 years in the TCGA dataset and 61.9 years in the validation dataset. The majority of patients in the TGCA dataset had stage II and III disease while in the validation dataset had stage II to IV disease.

**Table 2:** Clinical information of patients in the TCGA training dataset and GSE62254 validation dataset

Clinical characteristics	TCGA (N = 360)	GSE62254 (N = 300)
Age (years, mean ± sd)	64.9 ± 10.39	61.94 ± 11.36
Gender (male/female)	234/126	199/101
Pathologic_M (M0/M1/–)	328/18/14	273/27
Pathologic_N (N0/N1/N2/N3)	113/94/72/75/6	38/131/80/51
Pathologic_T (T1/T2/T3/T4/–)	17/70/167/105/1	2/186/91/21
Pathologic_stage (I/II/III/IV/–)	47/113/170/29/1	30/96/95/77/2
Targeted molecular therapy (yes/no/–)	144/193/23	–
Recurrence (yes/no)	77/253/30	125/157/18
Dead (death/alive/–)	122/238	135/148//17
Disease free survival (months, mean ± sd)	18.57 ± 17.19	33.72 ± 29.82
Overall survival time (months, mean ± sd)	16.17 ± 16.95	50.59 ± 31.42

“–” indicates the missing information

### Data normalisation and consistency selection

#### Data used for meta-analysis

Three of the five datasets used for meta-analysis, GSE29727, GSE54129, and GSE64951 were from the Affymetrix platform. The raw data from these three datasets was downloaded in the CEL format and were then transformed into gene symbols, their missing values were filled in using the median method, and then subjected to background correction using the minimal sets algorithm method and normalised using the quantiles method [10]. All of these methods were included in the oligo package from R (version 3.4.1, http://www.bioconductor.org/packages/release/bioc/html/oligo.html). The other two datasets, GSE26942 and GSE65801, were from the Illumina and Agilent platforms, respectively. These datasets were downloaded in TXT format and gene annotation was performed using the probe information provided by the platform. This data was then subjected to a logarithmic transformation and normalised using the quantiles method. These steps were performed using the limma package from R (version 3.4.1, https://bioconductor.org/packages/release/bioc/html/limma.html).

In the case of the gene methylation datasets, GSE25869 and GSE30601, the corresponding chromosome locations and methylated beta values were evaluated and assigned using the Methylation Module in GenomeStudio [PMID: 22498030] [11].

#### Quality control and consistency selection

Given the fact that these datasets were all derived from different platforms, meta-analysis was used to combine consistent data from across these datasets into a single larger dataset generating better statistical power and improving the reliability of the results. To eliminate potential bias, produced by differences in the platforms used to generate this data, all of the datasets were subjected to quality control using the criteria established in the MetaQC package from R (version 3.4.1, https://cran.r-project.org/web/packages/MetaQC/index.html). A total of five parameters, internal quality control (IQC), external quality control (EQC), accuracy quality control (AQC), consistency quality control (CQC), and standardised mean rank score (SMR), were calculated and evaluated. Then the reliable datasets were further analysed using the MetaDE.ES package (https://cran.r-project.org/web/packages/MetaDE) which selected the differentially expressed genes (DEGs) and differentially expressed methylation genes (DEMGs) when comparing GC and normal tissue samples. In brief, we performed a heterogeneity test for the expression of each gene on different platforms using tau^2^, Q value and Q pval as the measures. Then, we performed a heterogeneity test on the differential expression patterns for a gene in the integrated dataset across the different sample groups. Using this analysis we were able to determine the false discovery rate (FDR) which was validated via multiple-testing correction and we identified a value of < 0.05 as the significance threshold value for DEGs and DEMGs between different sample groups [12]. To ensure each signature gene exhibited consistent expression across different datasets, the thresholds for the homogeneity test were set as tau^2^ = 0 and Q pval > 0.05.

### Correlation analysis between gene expression and methylation

The datasets containing DEGs and DEMGs were selected and compared. Overlapping genes, those that were both differentially expressed and exhibited altered methylation levels, were identified using the cor function in R (version 3.4.1, http://127.0.0.1:19124/library/stats/html/cor.html). The Pearson coefficient (CC) for gene expression and methylation levels was calculated, and the genes with significant associations with the methylation data were selected as candidate signature genes. These signature genes were then subjected to gene oncology (GO) biological functional enrichment and Kyoto Encyclopedia of Genes and Genomes (KEGG, https://www.kegg.jp/) pathway enrichment analyses, using the Database for Annotation, Visualization and Integrated Discovery (DAVID, version 6.8, https://david.ncifcrf.gov/) by a hypergeometric distribution [13].

### Screening genetic prognostic biomarkers and clinical factors

Combining the identified tumour signature genes with the corresponding clinical factor information, we evaluated their correlation using univariate and multivariate cox regression analyses in the R survival package (version 3.4.1, http://bioconductor.org/packages/survivalr/). The threshold for significance was *P* < 0.05 when subjected to a log-rank test.

### Construction and validation of a predictive model of prognostic risk

#### Construction and validation of a tumour signature gene-based predictive model

Based on the prognostic information identified in the previous step, we produced a tumour signature gene-based predictive model which was then used to calculate the prognosis index (PI) value for each sample. The median PI score was set as the cut-off for classifying samples as high- or low-risk in the training dataset. Then, Kaplan–Meier (KM) survival curves were constructed using the survival package from R (version 3.4.1, http://bioconductor.org/packages/survival/) and used to evaluate the correlations between the predictive model and clinical outcome [14]. Meanwhile, these correlations were validated using the validation dataset. The area under the receiver operating characteristic (ROC) curve (AUROC) was used to determine the predictive accuracy of this model for both the training and validation datasets. The closer the AUROC value to 1.0 the higher the accuracy of the predictive model.

#### Construction of a prognostic clinical factor-based predictive model

We used a cox regression analysis to use the prognostic clinical factor information from each dataset to generate a clinical factor-based predictive model. In this model, the PI for each sample was calculated and the median value was set as the cut-off for the high- and low-risk groups from the training dataset. Likewise, the KM survival curve was used to assess the relationships between the predictive model and clinical prognosis. These results were also assessed using the validation dataset.

#### Construction of the predictive model integrating signature genes and clinical factors

We created a novel integrated prognostic model for GC by combining the prognostic results from the signature gene-based model with those from the clinical factor-based model. A new PI value was then calculated for each and the samples in the training dataset were divided into high- and low-risk groups using these new median values. KM survival curves were then used to evaluate the predictive value of these PI values and the results were evaluated in the validation dataset.

## Results

### Selection of DEGs and DEMGs

**Fig. 1:**
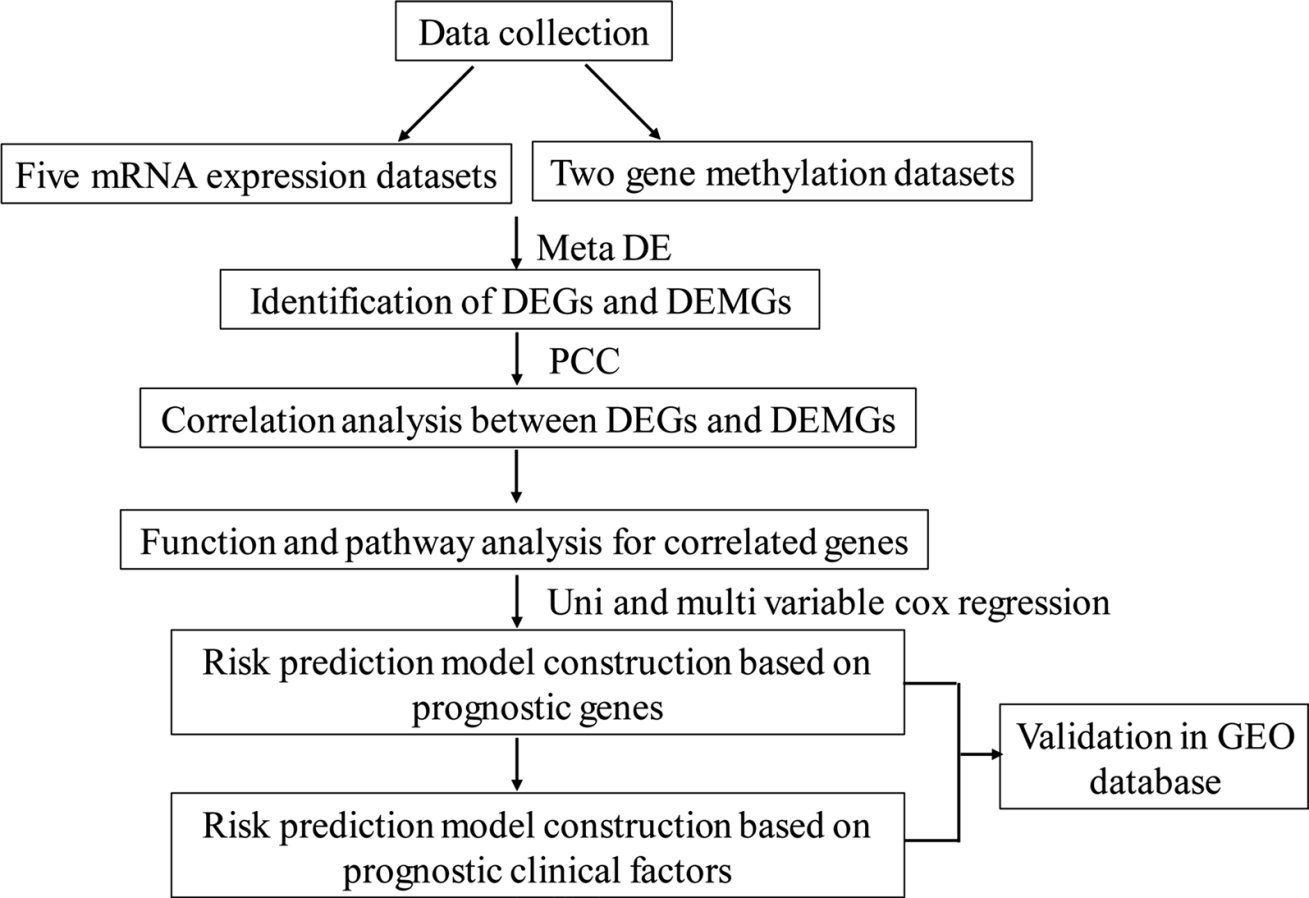
Flow chart describing the analytical process in this study. *DEG* differentially expressed gene, *DEMG* differentially expressed methylation genes, *PCC* Pearson coefficient correlation

After data normalisation, quality control of the datasets was conducted, and values for IQC, EQC, AQC, CQC, and SMR were calculated. The quality results indicated these datasets were all eligible for meta-analysis. Finally, we identified 1565 DEGs and 3754 DEMGs with a high degree of consistency between GC and normal samples, using MetaDE. The evaluation and identification process is described in Fig. 1.

### Correlation between gene expression and methylation

The DEGs and DEMGs were compared and matched, identifying 396 overlapping sequences that were differentially expressed at both the gene expression and methylation levels. We analysed the correlations between these values for each of the 396 genes identified from TCGA and GSE30601-GSE15460 (methylation profile with the matched gene profile) datasets. These evaluations indicated that overall gene expression was negatively associated with overall methylation in both TCGA (CC =  − 0.5145, *P* = 0.004) and GSE30601-GSE15460 (CC =  − 0.72704, *P* < 0.001) datasets. Given this, we then calculated the correlation values for gene expression and methylation for each gene, and genes with negative relationships, that is those genes with high degrees of methylation and low expression or vice versa, were retained. This evaluation narrowed our gene cohort to roughly 274 genes which were then evaluated as tumour signature genes.

Using GO functional and KEGG pathway enrichment analyses these 274 genes were found to be significantly enriched in 14 biological process categories including steroid metabolic process (*NR3C1*), fructose metabolic process (*FBP1*), regulation of cell migration (*VEGFC*), vitamin metabolic process (*ACADM*); and five pathway terms such as glycolysis/Gluconeogenesis (*FBP1*) (Table 3).

**Table 3:** Enrichment results of the candidate gene markers

Term	Count	*P* value	Genes
Biology process			
GO:0006929 ~ substrate-bound cell migration	4	6.96E−04	VEGFC, TNFRSF12A, ATP5B, MYH10
GO:0032101 ~ regulation of response to external stimulus	10	0.001453	EDNRA, GPX1, ADRB2, CYP27B1, ADORA2B, OSMR, FCER1G, GREM1, ADA, PLAU
GO:0051186 ~ cofactor metabolic process	11	0.001691	MTHFS, GPX1, ALAS1, HMBS, SUCLG1, MCCC1, ALDOB, GIF, UROD, PDHB, MDH1
GO:0050727 ~ regulation of inflammatory response	7	0.001717	EDNRA, GPX1, ADRB2, ADORA2B, OSMR, FCER1G, ADA
GO:0008202 ~ steroid metabolic process	11	0.002193	TM7SF2, OSBPL2, CYP27B1, SULT1B1, INSIG1, SCARB1, NR3C1, CAT, NR0B2, HSD17B8, FDFT1
GO:0016052 ~ carbohydrate catabolic process	8	0.002391	HYAL2, ALDOB, CHI3L1, FUT1, CTBS, PDHB, MDH1, ENO1
GO:0006091 ~ generation of precursor metabolites and energy	14	0.002446	NDUFB5, NDUFA9, KL, ATP5B, SUCLG1, FADS1, ALDOB, CRAT, PDHB, GFPT2, CAT, ENO1, ATP5J, MDH1
GO:0006000 ~ fructose metabolic process	4	0.002665	ALDOB, GFPT2, FBP1, FBP2
GO:0015980 ~ energy derivation by oxidation of organic compounds	9	0.002965	NDUFB5, KL, NDUFA9, SUCLG1, GFPT2, CRAT, CAT, PDHB, MDH1
GO:0006090 ~ pyruvate metabolic process	5	0.005245	ALDOB, FBP1, FBP2, PDHX, PDHB
GO:0030334 ~ regulation of cell migration	9	0.007713	PTPRK, VEGFC, MMP9, PTP4A1, RRAS2, SCARB1, GREM1, SST, ADA
GO:0006766 ~ vitamin metabolic process	6	0.008483	DHRS3, CYP27B1, ACADM, MCCC1, TMLHE, GIF
GO:0044271 ~ nitrogen compound biosynthetic process	13	0.008797	ATP5B, HMBS, ATP11B, PFAS, ADA, ADI1, ALAS1, TMLHE, NQO1, UROD, IMPDH1, ATP5J, ATP8A1
GO:0009310 ~ amine catabolic process	6	0.009967	MAOA, AMT, MCCC1, DDAH1, AUH, ENOSF1
KEGG pathway			
hsa00280:Valine, leucine and isoleucine degradation	6	0.003379	ACADM, IVD, OXCT1, MCCC1, PCCB, AUH
hsa05219:Bladder cancer	5	0.016087	RPS6KA5, VEGFC, CDKN1A, MMP9, CDK4
hsa03410:Base excision repair	4	0.047523	POLL, POLD1, NEIL1, PARP1
hsa00010:Glycolysis/Gluconeogenesis	5	0.045098	ALDOB, FBP1, FBP2, PDHB, ENO1
hsa00100:Steroid biosynthesis	3	0.045875	TM7SF2, CYP27B1, FDFT1

*GO* gene oncology, *KEGG*: Kyoto Encyclopedia of Genes and Genomes

### Prognostic gene biomarkers and clinical factors

**Fig. 2:**
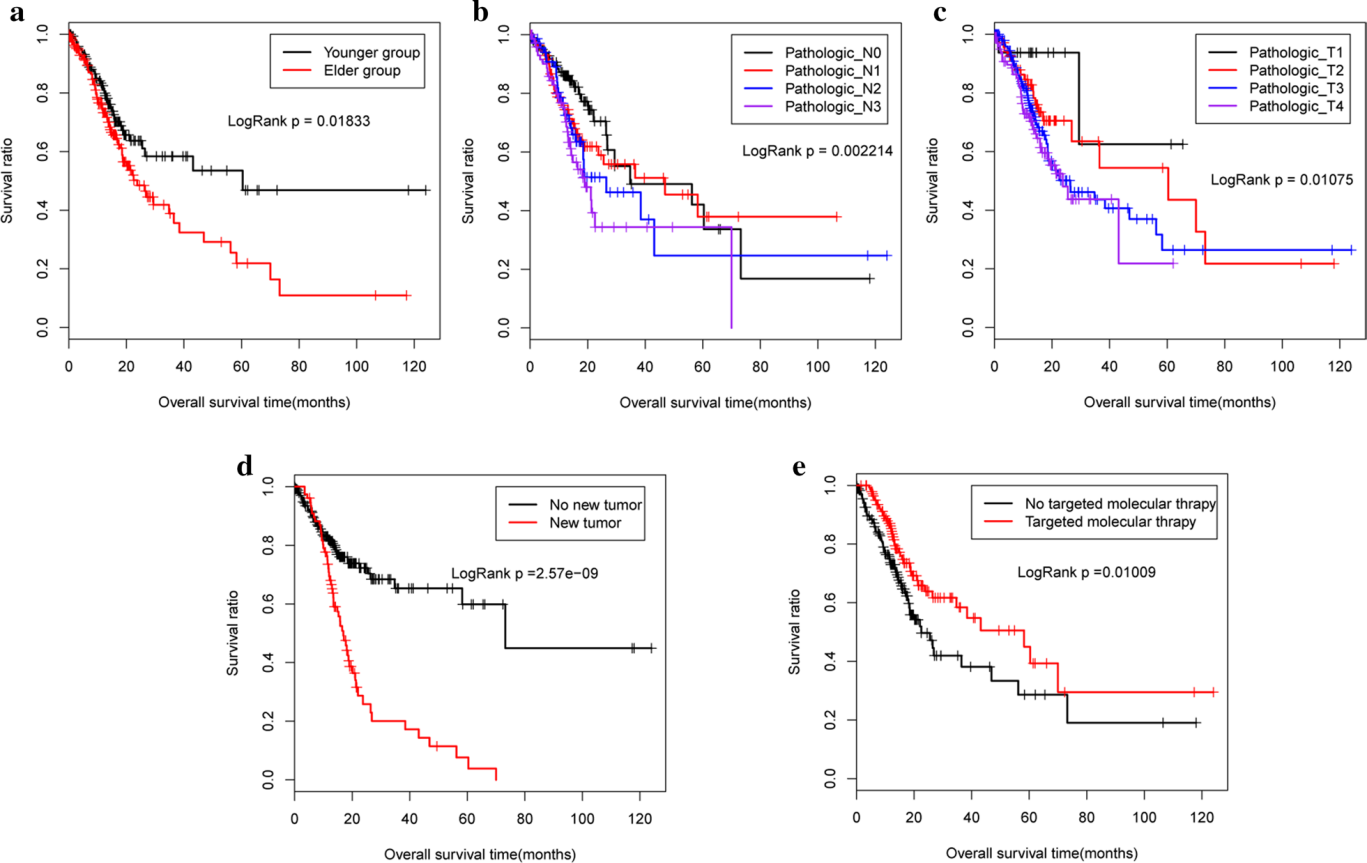
The Kaplan–Meier (KM) survival curves for five clinical factors. **a** age; **b** pathologic_N; **c** pathologic_T; **d** new tumour; **e** targeted molecular therapy

These 274 genes were then subjected to univariate and multivariate cox regression analyses to identify the prognostic genes and clinical factors. We finally selected 12 genes (*SLC5A5*, *SLC7A6*, *NFE2L2*, *DFNA5*, *VEGFC*, *MUM1*, *TRIB2*, *MCOLN1*, *FBP1*, *ACADM*, *WDR37*, and *NR3C1*) that demonstrated a significant correlation with clinical prognosis (Table 4), and five independent clinical factors (age, pathologic_N, pathologic_T, targeted molecular therapy, and new tumour) for our predictive models (Table 5). The KM survival curves for each are shown in Fig. 2.

**Table 4:** Gene markers significantly related to the prognosis

Gene	Coefficient correlation	Hazard ratio	*P* value
SLC5A5	0.441439	1.5549	1.35E−06
SLC7A6	0.693078	1.9999	0.001005
NFE2L2	– 0.656704	0.5186	0.010575
DFNA5	0.371722	1.4502	0.010885
VEGFC	0.647272	1.9103	0.015025
MUM1	– 0.664478	0.5145	0.015945
TRIB2	0.321468	1.3792	0.02099
MCOLN1	– 0.593416	0.5524	0.02702
FBP1	– 0.218411	0.8038	0.033745
ACADM	– 0.374113	0.6879	0.037275
WDR37	– 0.627658	0.5338	0.038765
NR3C1	– 0.40384	0.6678	0.0455

**Table 5:** Clinical factors identified using cox regression analysis

Clinical characteristics	Univariate cox regression		Multivariate cox regression	
	*P* value	HR (95%CI)	*P* value	HR (95%CI)
Gender (male/female)	0.05828	1.468 (0.984–2.19)	0.10378	1.4823 (0.9226–2.3818)
Pathologic_M (M0/M1/–)	0.004448	2.495 (1.3–4.788)	0.06341	2.3737 (0.9529–5.9131)
Pathologic_stage (I/II/III/IV/–)	9.24E−05	1.567 (1.249–1.967)	0.92892	0.9803 (0.6325–1.5193)
Radiation therapy (Yes/No/–)	3.35E−03	0.4701 (0.281–0.7865)	0.62836	0.8449 (0.4271–1.6717)
Age (above/below median (65))	0.01833	1.556 (1.074–2.252)	0.04577	1.5779 (1.0086–2.4685)
Pathologic_N (N0/N1/N2/N3/–)	0.002214	1.284 (1.092–1.509)	0.0364	1.3105 (1.0173–1.6883)
Pathologic_T (T1/T2/T3/T4/–)	0.01075	1.345 (1.07–1.691)	0.01735	1.4999 (1.0741–2.0946)
Targeted molecular therapy (yes/no/–)	0.01009	0.609 (0.4158–0.8919)	0.00279	0.4432 (0.2600–0.7555)
New tumor (yes/no/–)	2.57E−09	2.976 (2.042–4.338)	9.21E−08	3.1742 (2.0777–4.8494)

**Fig. 3:**
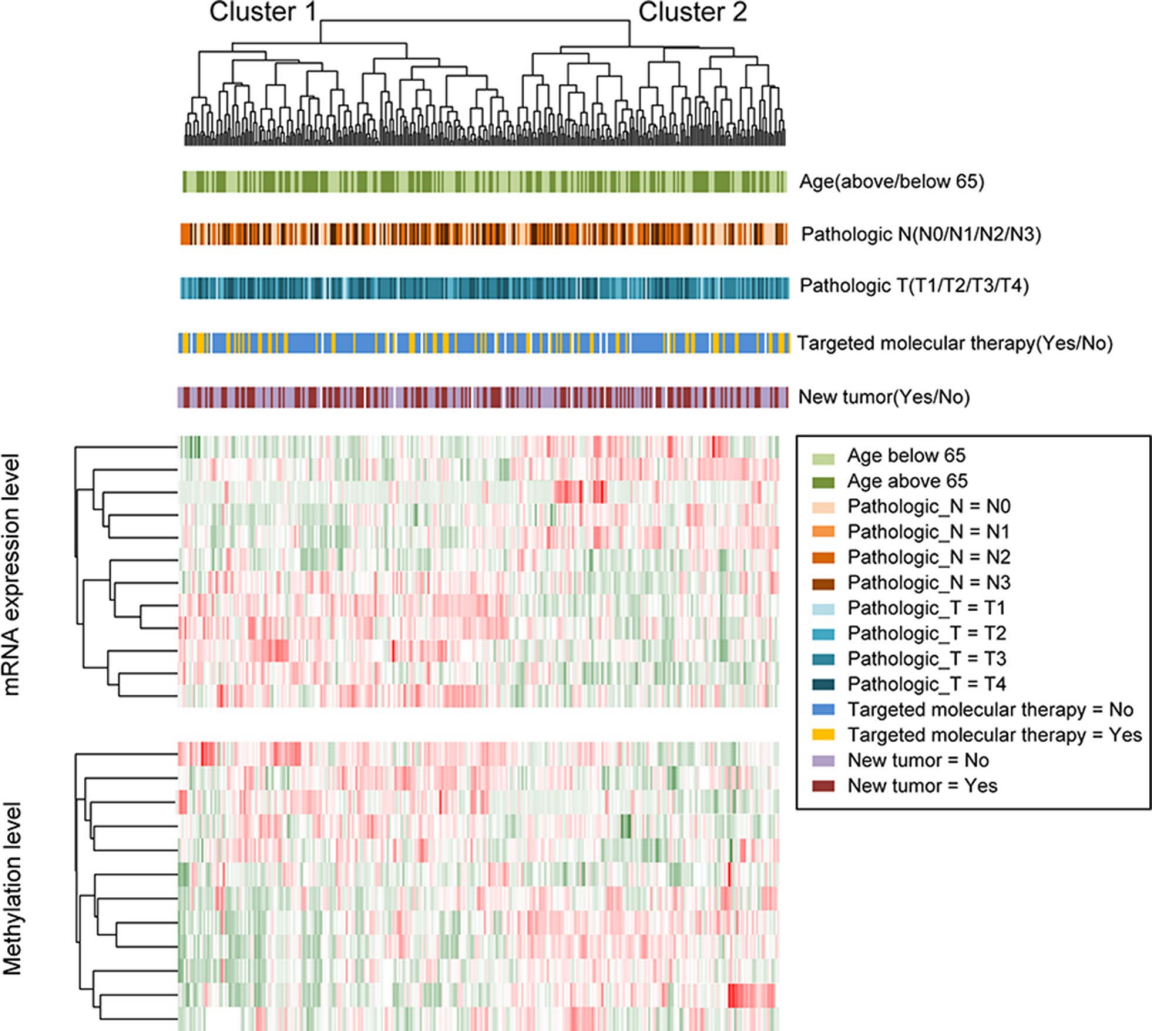
Clustered heatmap describing the gene expression and methylation patterns of the 12 signature genes and their correlation with specific prognostic clinical factors

We developed a clustering heatmap showing the expression and methylation status of all 12 of our tumour signature genes and then combined these with their corresponding prognostic clinical factors (Fig. 3). This analysis revealed that four clinical factors, age, pathologic_N, targeted molecular therapy, and new tumour, were significantly associated with the prognosis of patients in the two clusters (*P* < 0.01).

### Construction and validation of an integrated prognostic risk prediction model

#### The signature gene-based risk predictive model

After obtaining the prognostic gene information using the cox regression algorithm, we constructed a prognostic risk prediction model using our 12 signature genes. The samples in the training dataset were classified as high- or low-risk with their cut-off set to the median PI values.

**Fig. 4:**
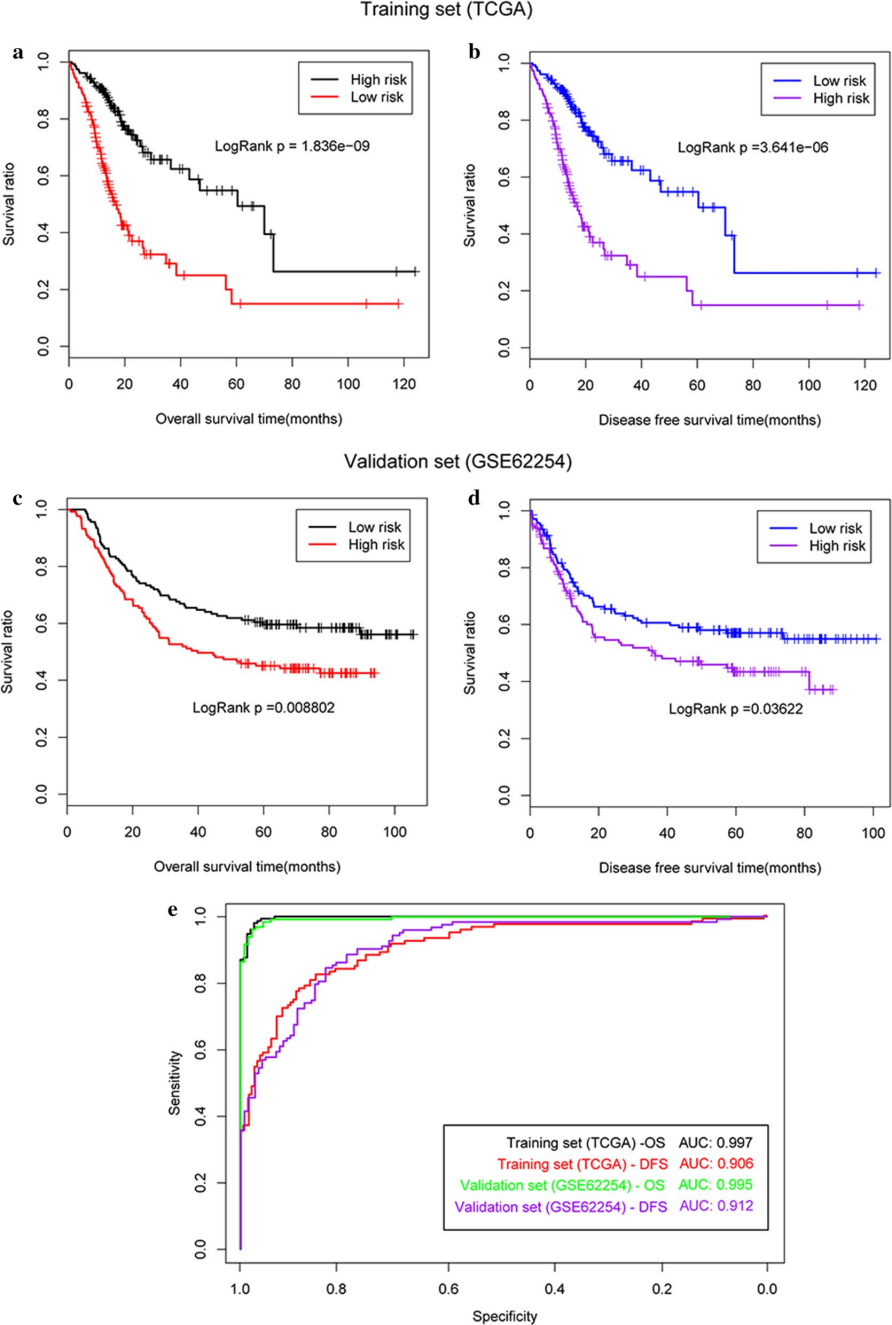
Survival curves generated using the gene-based predictive model. **a** Overall survival (OS) of patients from TCGA dataset; **b** disease free survival (PFS) of patients from TCGA; **c** OS of patients from the validation dataset; **d** DFS of patients from the validation dataset; **e** the area under the ROC (AUROC) for the survival curves from both TCGA and validation datasets

In the training dataset, the survival analysis indicated that patients in the low-risk group had a significantly longer median overall survival (OS) (22.1 m vs. 15.1 m, *P* < 0.001, Fig. 4a) and median disease free survival (DFS) (22.1 m vs. 14.5 m, *P* < 0.001, Fig. 4b), than those patients in the high-risk group. The AUROC for the OS and DFS curves were 0.997 and 0.906, respectively (Fig. 4e), suggesting that both had a high predictive accuracy.

Similarly, in the validation dataset GSE62254, patients in the low-risk group had better survival rates compared with those in the high-risk group as evidenced by a prolonged median OS (55.8 m vs. 44.2 m, *P* = 0.009, Fig. 4c) and DFS (41.6 m vs. 30.6 m, *P* = 0.036, Fig. 4d) value. The AUROCs for these two outcomes were 0.995 and 0.912, respectively (Fig. 4e), indicating that this model created values with high predictive accuracy.

#### The clinical factor-based prognostic risk model

Five clinical factors were identified using a cox regression algorithm, and based on the weight of their coefficients, samples containing all five factors were selected (n = 283) to establish the clinical factor-based predictive model for prognostic risk. The PI of each sample was calculated and then used to classify the samples.

**Fig. 5:**
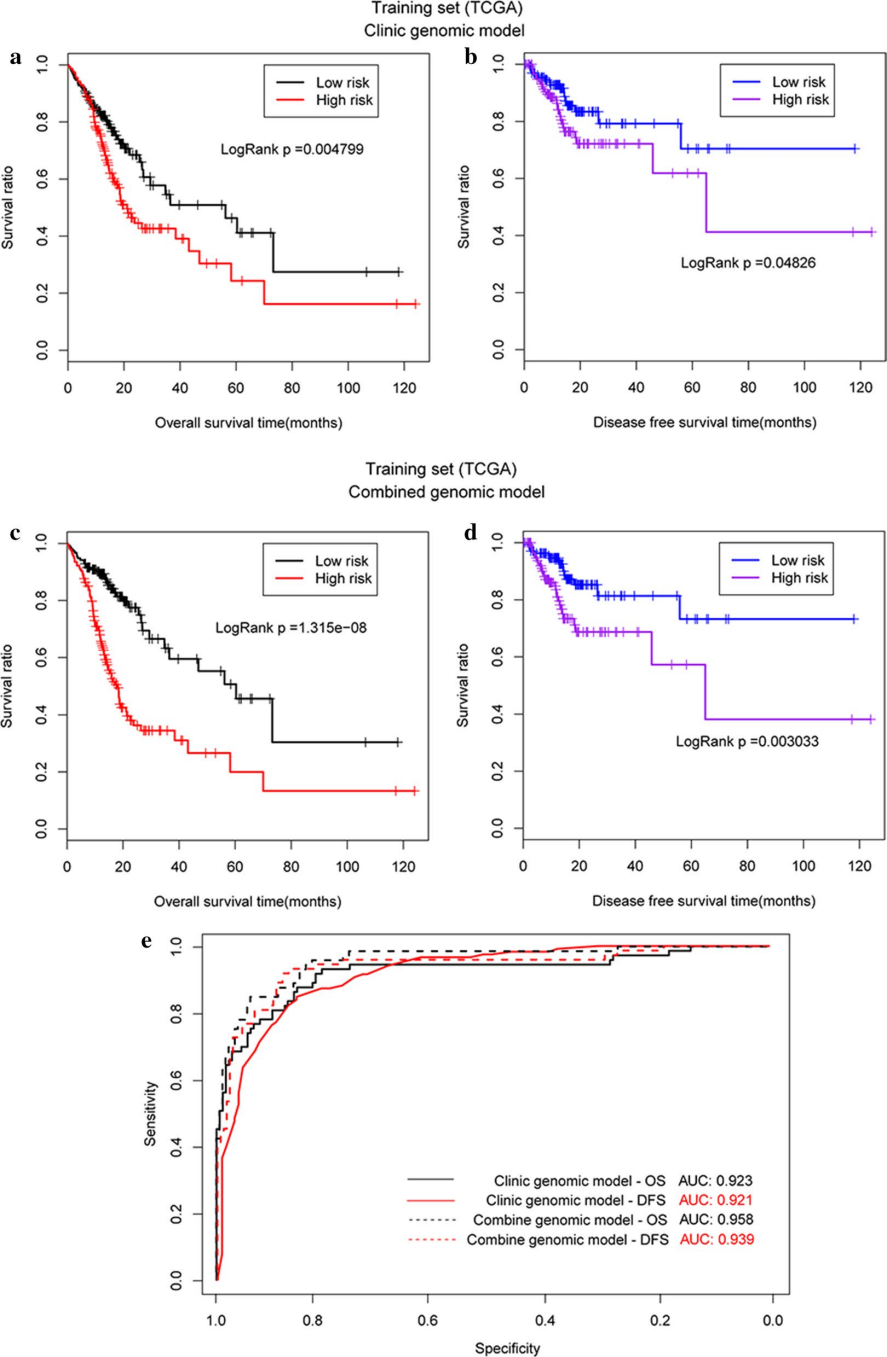
Survival curves generated using the prognostic clinical factor-based predictive model. **a** OS of patients from TCGA dataset; **b** DFS of patients from TCGA; **c** OS of patients from the validation dataset; **d** DFS of patients from the validation dataset; **e** AUROC for each of the survival curves from both the TCGA and validation datasets

Survival analysis showed that the OS (19.5 m vs. 16.6 m, *P* = 0.005, Fig. 5a) and DFS (18.5 m vs. 17.8 m, *P* = 0.048, Fig. 5b) of patients were significantly prolonged in the low-risk group when compared with those of the high-risk group. The AUROC was determined to be 0.923 and 0.921, respectively (Fig. 5e).

In the GSE62254 validation dataset, only three clinical factors, age, pathologic_N, and pathologic_T, were available. Thus, we adapted the three clinical factor-based predictive model to build a clinical factor-based model, to validate the results derived in TCGA training dataset. We again showed that patients in the low-risk group had significantly prolonged OS (58.9 m vs. 37.2 m, *P* < 0.0001, Fig. 5c) and DFS (44.4 m vs. 24.3 m, *P* < 0.0001, Fig. 5d) compared to patients in the high-risk group. The AUROC values for OS and DFS were 0.897 and 0.882, respectively (Fig. 5e).

#### Building an integrated predictive model incorporating both clinical factors and signature gene expression

The integrated predictive model was constructed by combining the weight coefficient from the 12 signature genes and five clinical factors. Then, the PI of each sample was re-calculated, and then reassigned as low- or high-risk.

**Fig. 6:**
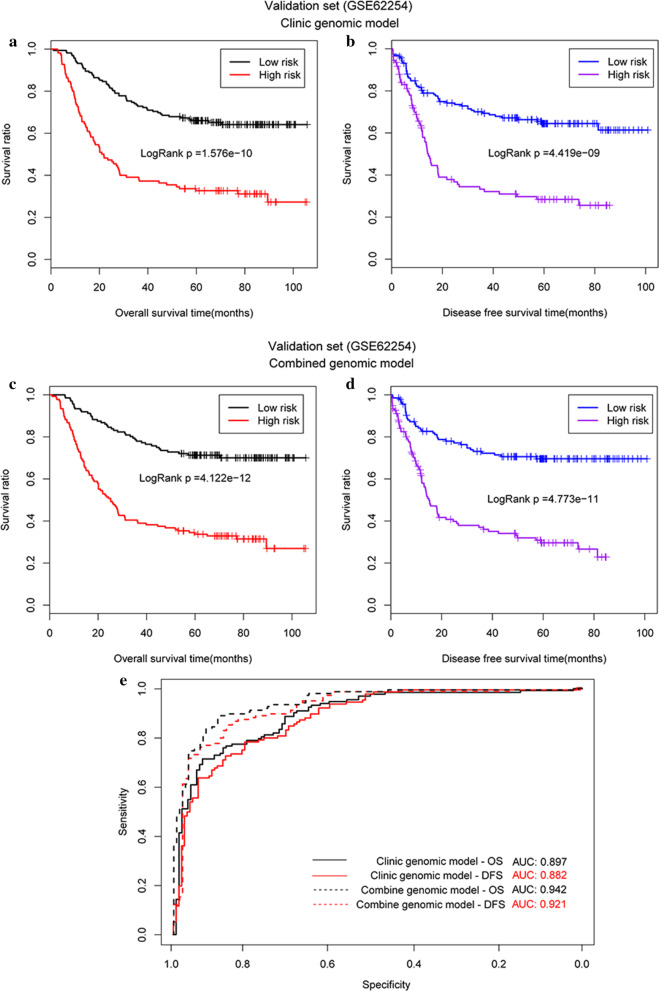
Survival curves generated using the integrated predictive. **a** OS of patients from the TCGA dataset; **b** DFS of patients from TCGA; **c** OS of patients from the validation dataset; **d** DFS of patients from the validation dataset; **e** AUROC values for the survival curves generated from both the TCGA and validation datasets

The patients in TGCA dataset low-risk group had a significantly prolonged OS (20.3 m vs. 15.8 m, *P* < 0.001, Fig. 6a) and DFS (19.7 m vs. 14.6 m, *P* = 0.003, Fig. 6b) compared with those in the high-risk group. The AUROCs of the two outcomes were 0.985 and 0.939, respectively (Fig. 6e).

To evaluate the GSE62254 validation dataset, the clinical factors were reduced from five to three based on data availability and new PI values were calculated. Patients were then categorised as high or low risk and then their survival was evaluated. Patients in the low-risk group were shown to have significantly longer survival rates than patients in the high-risk group (OS: 62.3 m vs. 38.0 m, *P* < 0.0001, Fig. 6c; DFS: 47.9 m vs. 24.6 m, *P* < 0.0001, Fig. 6d). The AUROC values for OS and DFS were 0.942 and 0.921, respectively (Fig. 6e).

## Discussion

Here, we used a series of bioinformatics analyses to construct a predictive model for evaluating the prognosis of GC samples using 12 signature genes and five clinical factors. All 12 signature genes were also differentially methylated and could be used to split patients into high and low risk groups. These models were all validated using a validation set.

When we evaluated our 12 signature genes, four were identified as particularly interesting, vascular endothelial growth factor c (*VEGFC*), nuclear receptor subfamily 3 group c member 1 (*NR3C1*), nuclear factor, erythroid 2 like 2 (*NFE2L2*), and fructose-1,6-bisphosphatase-1 (*FBP1*). *VEGFC* has been reported to be a key regulator in GC progression and its encoded protein facilitates angiogenesis and endothelial cell growth. In addition, oxidised low-density lipoprotein (oxLDL) is a risk factor in the pathogenesis of cancers linked to its roles in abnormal lipid metabolism, and has been shown to promote lymphatic metastasis of GC via the up-regulated expression and secretion of *VEGFC* [15]. microRNA (miR)-27b acts as a potential tumour suppressor in GC and targets *VEGFC* expression [16], while miR-101 promotes cisplatin (DPP)-induced apoptosis partly via its targeting of *VEGFC* in DDP-resistant GC cells [17]. In addition, *VEGFC* expression is associated with the GC prognosis, as survival is significantly poorer in *VEGFC*-positive GC patients, when compared to *VEGFC*-negative patients [18]. Moreover, decreased *VEGFC* was shown to correlate with an increased risk of tumour progression [19]. Here, we identified *VEGFC* as one of the 12 signature genes for evaluating GC prognosis and this gene was enriched in the ‘regulation of cell migration’ functional category which suggests that *VEGFC* methylation may be related to GC prognosis via its regulation of cell migration. However, this regulatory relationship needs to be further validated in vitro and across large populations.

FBP1 protein is a gluconeogenesis regulatory enzyme associated with metabolic acidosis. Snail is an important mediator in cancer and has been shown to be increased in GC inducing the glucose metabolism via the down-regulated expression of *FBP1* [20] indirectly regulating the epithelial-mesenchymal transition (EMT). Decreased *FBP1* serves as a positive factor in the metastasis of GC and is an indicator of poor prognosis in patients [21]. In GC cell lines, *FBP1* is downregulated and its promoter is hypermethylated, resulting in increased carcinogenesis. Moreover, the methylation of *FBP1* at its promoter has been independently associated with GC prognosis [22]. This was consistent with our findings that *FBP1* was identified as one of the 12 signature genes having some predictive value for GC prognosis. Additionally, this gene was enriched in glycometabolism-related functions and pathways. When taken collectively these data indicated that DNA methylation of *FBP1* may be associated with GC prognosis via the differential regulation of the glycometabolism.

The *NR3C1* gene encodes a glucocorticoid receptor. *NR3C1* is important in the carcinogenesis of GC and has been used as a marker to identify primary GC [23, 24]. The high degree of methylation within the *NR3C1* promoter was also implicated in the initiation of GC progression, and four SNPs at this locus have been shown to be strongly associated with increased risk for GC in a Chinese population [20]. Here, we confirmed the link between *NR3C1* methylation and GC prognosis, and suggest that *NR3C1* methylation may be a reliable prognostic indicator for GC.

*NFE2L2*, also known as *NRF2*, encodes a transcription factor (TF) known to participate in GC development, and its overexpression is a predictive marker for the prognosis and 5-FU resistance in GC [25]. GC patients positive for *NRF2* expression are known to exhibit significantly poorer OS rates when compared to *NRF2*-negative patients [26]. Deafness associated tumour suppressor (*DFNA5*) is inactivated in GC via methylation, and this methylation is found in half of all patients with primary GC [27]. Here, we propose that there is a relationship between the methylation status of *NFE2L2* and *NRF2* and the prognosis of GC.

Finally, our analysis suggests that the predictive models produced in this study were relatively precise probably as a result of the increased sample size resulting from our meta-analysis. In addition, to relatively high AUROC values, our predictive models provided reliable results in our validation datasets. These encouraging results shed lights on potential regulatory mechanisms on methylation genes in GC prognosis. In addition, this pilot bioinformatics analysis will lay the foundation of exploratory biomarker analysis, which could facilitate to the prediction or indication of patients with a low risk of death and a good survival outcome. Importantly, by the identification of these sensitive methylation gene markers and the methylation patterns, we might have a deeper understanding on this malignancy progression and might develop novel targeted therapies, which could improve the survival outcomes of the patients with GC. However, several limitations remain. The expression and methylation of these signature genes should be validated in vitro and in vivo with substantial cell lines and animal samples. Moreover, perspective studies are warranted using larger clinical cohorts to validate the prognostic values of these genes before being adopted in diagnostic and prognostic settings, and we will perform these studies in future.

## Conclusion

In conclusion, methylation of 12 signature genes, including *VEGFC*, *FBP1*, *NR3C1*, *NFE2L2*, and *DFNA5*, may be associated with the prognosis of GC, and these genes-based risk models may be a useful tool in predicting prognostic outcomes for patients at earlier stages of disease. However, these results require validation in larger patient cohorts before they can be confidently applied in a clinical setting.

## Abbreviations

GC: Gastric cancer
TCGA: The Cancer Genome Atlas
IQC: Internal quality control
EQC: External quality control
AQC: Accuracy quality control
CQC: Consistency quality control
SMR: Standardised mean rank score
DEGs: Differentially expressed genes
DEMGs: Differentially expressed methylation genes
FDR: False discovery rate
CC: Coefficient correlation
GO: Gene oncology
KEGG: Kyoto Encyclopedia of Genes and Genomes
PI: Prognosis index
KM: Kaplan–Meier
ROC: Receiver operating characteristic
AUROC: Area under the ROC
VEGFC: Vascular endothelial growth factor c

## Availability of data and material

The raw data were collected from the GEO public database, GSE26942 was from the GPL6947 platform (https://www.ncbi.nlm.nih.gov/geo/query/acc.cgi?acc=GSE26942); GSE29727 was isolated from GPL96 platform (https://www.ncbi.nlm.nih.gov/geo/query/acc.cgi?acc=GSE29727); GSE54129 (https://www.ncbi.nlm.nih.gov/geo/query/acc.cgi?acc=GSE54129) and GSE64951 (https://www.ncbi.nlm.nih.gov/geo/query/acc.cgi?acc=GSE64951) were both from GPL570. GSE65801 was from the GPL14550 (https://www.ncbi.nlm.nih.gov/geo/query/acc.cgi?acc=GSE65801); the methylation datasets of GSE25869 (https://www.ncbi.nlm.nih.gov/geo/query/acc.cgi?acc=GSE25869) and GSE30601 (https://www.ncbi.nlm.nih.gov/geo/query/acc.cgi?acc=GSE30601) were identified from the GPL8490 platform. The relative gastric cancer gene expression and methylation profiles were downloaded from The Cancer Genome Atlas (TCGA, https://gdc-portal.nci.nih.gov/) database. The gastric cancer-related gene expression profile in GSE62254 was downloaded from the GEO database (https://www.ncbi.nlm.nih.gov/geo/query/acc.cgi?acc=GSE62254).
